# Extracellular Superoxide Dismutase Expression in Papillary Thyroid Cancer Mesenchymal Stem/Stromal Cells Modulates Cancer Cell Growth and Migration

**DOI:** 10.1038/srep41416

**Published:** 2017-02-20

**Authors:** Alessia Parascandolo, Francesca Rappa, Francesco Cappello, Jaehyup Kim, David A. Cantu, Herbert Chen, Gianluigi Mazzoccoli, Peiman Hematti, Maria Domenica Castellone, Marco Salvatore, Mikko O. Laukkanen

**Affiliations:** 1 IRCCS SDN, Naples 80143, Italy; 2 Department of Experimental and Clinical Neurosciences, University of Palermo, 90133, Italy; 3 Euro-Mediterranean Institute of Science and Technology (IEMEST), Palermo, Italy; 4 Department of Medicine, University of Wisconsin, Madison, Wisconsin 53792, USA; 5 Department of Surgery, University of Alabama, Birmingham, AL, USA; 6 Department of Medical Sciences, Division of Internal Medicine and Chronobiology Unit, IRCCS “Casa Sollievo della Sofferenza”, S. Giovanni Rotondo (FG) 71013, Italy; 7 University of Wisconsin Carbone Cancer Center, Madison, Wisconsin 53792, USA; 8 Institute of Experimental Endocrinology and Oncology (CNR), 80014 Naples, Italy; 9 Department of Molecular Medicine and Medical Biotechnologies, University of Naples Federico II, 80014 Naples, Italy

## Abstract

Tumor stroma-secreted growth factors, cytokines, and reactive oxygen species (ROS) influence tumor development from early stages to the metastasis phase. Previous studies have demonstrated downregulation of ROS-producing extracellular superoxide dismutase (SOD3) in thyroid cancer cell lines although according to recent data, the expression of SOD3 at physiological levels stimulates normal and cancer cell proliferation. Therefore, to analyze the expression of SOD3 in tumor stroma, we characterized stromal cells from the thyroid. We report mutually exclusive desmoplasia and inflammation in papillary and follicular thyroid cancers and the presence of multipotent mesenchymal stem/stromal cells (MSCs) in non-carcinogenic thyroids and papillary thyroid cancer (PTC). The phenotypic and differentiation characteristics of Thyroid MSCs and PTC MSCs were comparable with bone marrow MSCs. A molecular level analysis showed increased *FIBROBLAST ACTIVATING PROTEIN, COLLAGEN 1 TYPE A1, TENASCIN*, and *SOD3* expression in PTC MSCs compared to Thyroid MSCs, suggesting the presence of MSCs with a fibrotic fingerprint in papillary thyroid cancer tumors and the autocrine-paracrine conversion of *SOD3* expression, which was enhanced by cancer cells. Stromal SOD3 had a stimulatory effect on cancer cell growth and an inhibitory effect on cancer cell migration, thus indicating that SOD3 might be a novel player in thyroid tumor stroma.

In solid tumors, paracrine factors secreted from the stroma regulate cancer cell growth and migration^1^,^2^,^3^,^4^,^5^,^6^,^7^,^8^,^9^. Reactive oxygen species (ROS), a well-known paracrine factor, contribute to stromal myofibroblast maturation^10^, thus emphasizing the effect of ROS in tumorigenesis. Extracellular superoxide dismutase (SOD3) has anti-oxidative, anti-inflammatory, anti-apoptotic, and growth promoting characteristics, exhibiting the most potent therapeutic responses and growth regulatory characteristics in cardiovascular and cancer models^11^,^12^,^13^,^14^,^15^,^16^,^17^,^18^,^19^,^20^,^21^,^22^. The expression of *SOD3* is increased in a benign thyroid tumor goiter model and gradually downregulated in cell lines that model advanced papillary and anaplastic thyroid cancers correlating with the level of oncogene activation^23^,^24^. Of note, downregulation of growth stimulating *SOD3* in epithelial cancer cells is controversial, particularly in light of recent data demonstrating SOD3-driven immortalization and even the transformation of murine primary cells, hence suggesting abrogation of the growth advantage in cancer cells^23^,^24^,^25^,^26^,^27^,^28^.

In the current study, we describe mesenchymal stem cells (MSCs) isolated from non-carcinogenic thyroids (Thyroid MSCs) and papillary thyroid cancer (PTC MSCs), the latter showing desmoplastic characteristics. Importantly, a redox gene expression analysis showed downregulation of *SOD3* in papillary thyroid cancer TPC1 cells compared to Nthy control cells and upregulation in PTC MCS compared to Thyroid MSCs, hence suggesting autocrine-paracrine conversion of *SOD3* mRNA expression. A functional analysis of stromal secreted SOD3 corroborated previously published data^20^,^26^ showing increased cancer cell proliferation and decreased cell migration in co-culture. Therefore, our data suggest that the growth-promoting characteristics of SOD3 are not limited to the initial benign growth phase of tumorigenesis but are sustained to the end phase of tumor development.

## Results

### Histological analysis of papillary thyroid cancer and follicular thyroid cancer stroma sections

**Figure 1:**
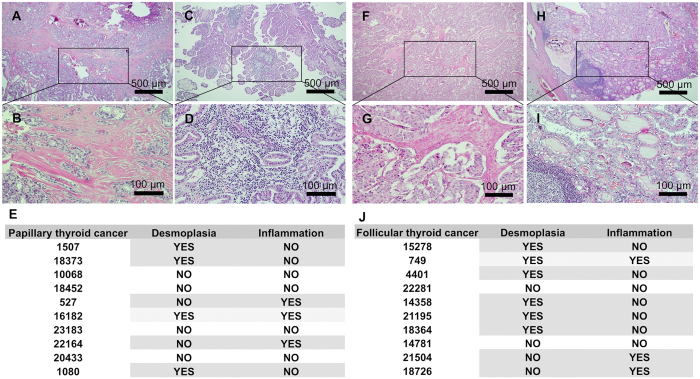
Representative histological images of hematoxylin-eosin staining of sections from papillary (**A**–**D**) and follicular (**F**–**I**) thyroid cancer. (**A**,**B**) Papillary thyroid cancer regions with desmoplastic stroma. (**C**,**D**) Papillary thyroid cancer regions with mononuclear cell infiltration. (**E**) Table showing papillary thyroid cancer patient numbers and the corresponding desmoplasia and/or inflammation. (**F**,**G**) Follicular thyroid cancer regions with desmoplastic stroma. (**C**,**D**) Follicular thyroid cancer regions with mononuclear cell infiltration. (**E**) Table showing follicular thyroid cancer patient numbers and the corresponding desmoplasia and/or inflammation. Calibration bars: 500 μm (**A**,**C**,**F**,**H**); 100 μm (**B**,**D**,**G**,**I**).

In thyroid cancers, desmoplastic stromal reactions, which correlate to lymph node metastasis, are a relatively common early phenomenon present in up to 80% of medullary thyroid cancers^29^. Characterization of papillary (PTC) and follicular (FTC) thyroid cancers 12 out of 20 cases (60%) demonstrated fibrosis or mononuclear cell infiltration. In PTC 40% of tumors showed desmoplastic regions and 30% inflammatory regions, whereas 40% of PTC tumors showed no detectable changes in stroma. In one case (10%) the stroma contained both desmoplastic and inflammatory regions. Interestingly, 50% of the cases suggested mutual exclusion between fibrosis and inflammation (Fig. 1A–D and F–I). The analysis of FTC showed desmoplasia or mononuclear cell infiltration in 8 out of 10 cases (80%). In seven cases (70%) there was mutual exclusion between fibrosis and inflammation: in five cases (50%) there was desmoplasia without inflammation and in two cases (20%) there was increased inflammation without fibrosis. In two cases (20%) there was no desmoplasia or inflammation, and in one case (10%) FTC stroma showed both desmoplasia and increased mononuclear cell content (Fig. 1E,J).

### Mesenchymal stem cells from thyroid and papillary thyroid cancer

**Figure 2:**
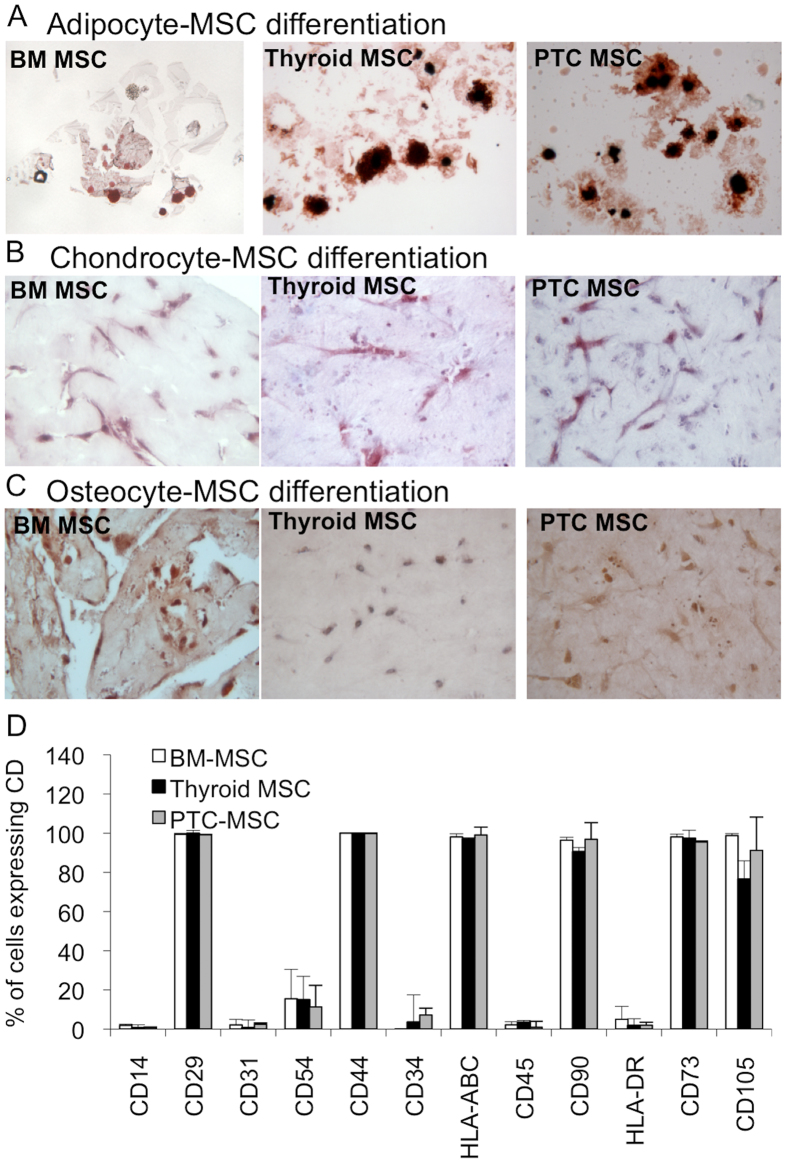
Phenotypic assays for isolated BM MSCs, Thyroid MSCs, and PTC MSCs. (**A**) Adipocyte, (**B**) chondrocyte, and (**C**) osteocyte differentiation assays suggested no differences between BM-derived and thyroid-derived MSCs. (**D**) Flow cytometer analysis supported the differentiation data showing identical of cluster of differentiation (CD) expression in BM MSC, Thyroid MSC, and PTC MSC cell surfaces.

Most of the tissues have been suggested to contain multipotent mesenchymal stem/progenitor cells that support tissue renewal and function as a source of cytokines and growth factors^30^. To study the presence of MSCs in papillary thyroid cancer and a non-carcinogenic thyroid tissue counterpart, we isolated plastic adherent mesenchymal cells and characterized their phenotype. To test the stemness of the isolated cells, adipocyte, chondrocyte, and osteocyte lineage differentiation assays were performed to define the multipotency of the isolated cells. We observed similar multipotency among the bone marrow derived MSCs (BM MSC), the Thyroid MSCs, and the PTC MSCs, indicating the presence of mesenchymal stem cells in the adult thyroid tissues and papillary thyroid cancer tumor tissues (Fig. 2A–C). The analysis of the expression of cell surface cluster of differentiation (CD) molecules used to identify MSCs by flow cytometer suggested no differences among the BM MSCs, Thyroid MSCs, and PTC MSCs (Fig. 2D), corroborating the differentiation data and suggesting the presence of mesenchymal stem cells similar to BM MSCs in thyroid and thyroid cancers.

### PTC MSCs have fibrotic characteristics

**Figure 3:**
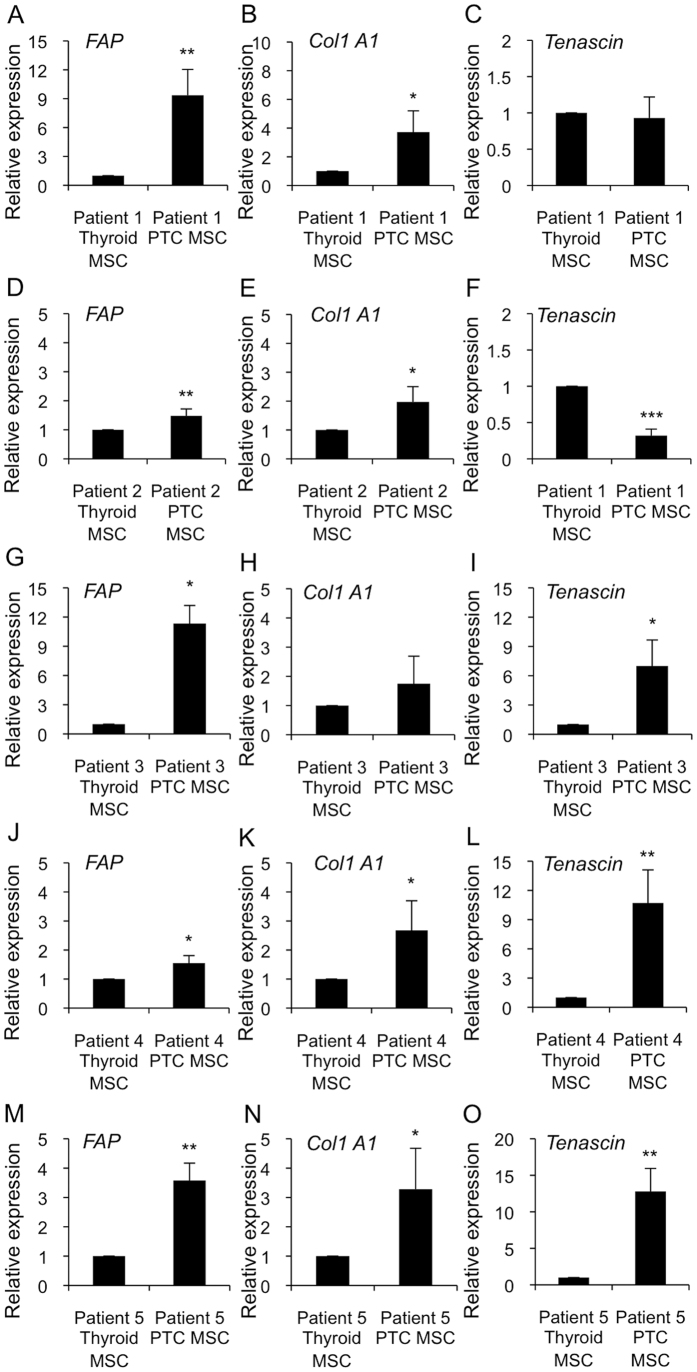
Expression analysis of *FAP, Col1A1*, and *TENASCIN* desmoplastic markers in Thyroid MSCs and PTC MSCs. (**A**–**O**) The qRT-PCR data demonstrated significantly increased desmoplastic marker expression in all five patient-derived PTC MSC samples compared to their non-carcinogenic thyroid counterparts. (**A**–**C**) Patient 1, (**D**–**F**) Patients 2, (**G**–**I**) Patient 3, (**J**–**L**) Patient 4, and (**M**–**O**) Patient 5. The p-values (*p < 0.05, **p < 0.01, ***p < 0.001) were determined by two-tail independent samples t-tests.

Because our data showed the presence of fibrosis in papillary thyroid cancer, we studied desmoplastic molecular marker expression from Thyroid MSCs and PTC MSCs. Strikingly, in all five patients, PTC MSCs had higher *FIBROBLAST ACTIVATING PROTEIN (FAP*) mRNA expression compared to Thyroid MSCs. Similarly, PTC MSCs had significantly higher *COLLAGEN 1 TYPE A1 (Col1 A1*) and *TENASCIN* mRNA expression levels as compared to Thyroid MSCs in four out of five and in three out of five patients, respectively. Although we observed variation in fibrotic marker expression in the patient 1 (*TENASCIN* expression was at the level of controls), in the patient 2 (*TENASCIN* expression lower than in the controls), and in the patient 3 (*Col1 A1* expression was at the level of controls) our data could suggest that mesenchymal stem cells may function as an origin for myofibroblasts (Fig. 3A–O).

### *SOD3* expression is reduced in epithelial cancer cells and is increased in MSCs with fibrotic characteristics

**Figure 4:**
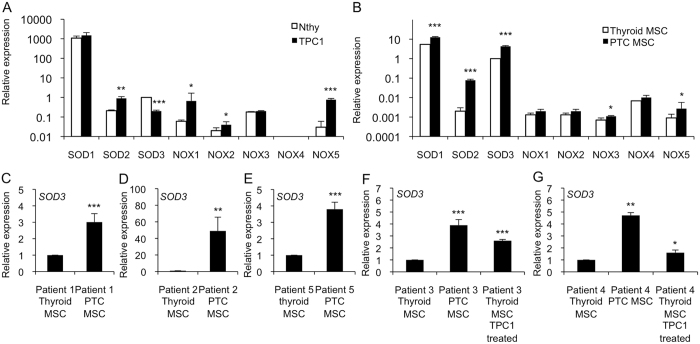
Gene expression of *SOD1-3* and *NOX1-5* in thyroid cells. We used Nthy cells modeling normal thyroid epithelia, TPC1 cells modeling papillary thyroid cancer, Thyroid MSCs, and PTC MSCs. (**A**) *SOD1, SOD2, NOX1-3* and *NOX5* showed increased expression levels in TPC1 cells compared to in the Nthy cell line, whereas *SOD3* expression was downregulated in the TPC1 cells. *NOX4* expression was not detectable. (**B**) *SOD1, SOD2, NOX1-3*, and *NOX5* expression levels in the PTC MSCs were increased or remained at the same level as in the Thyroid MSCs. *SOD3* expression was significantly higher in the PTC MSCs compared to in the Thyroid MSCs. There was high *NOX4* expression in both the Thyroid MSCs and the PTC MSCs. (**C**–**G**) Increased *SOD3* mRNA synthesis was confirmed in each of the five patients. (**F**,**G**) Expression analysis using TPC1-condensed medium suggested a cancer-derived paracrine effect in SOD3 activation. The p-values (*p < 0.05, **p < 0.01, ***p < 0.001) were determined by two-tail independent samples t-tests.

Thyroid cancer progression and fibrosis correlate with increased reactive oxygen species (ROS) production and increased redox enzyme expression^23^,^31^. Hence, we investigated the gene expression of the superoxide dismutase *SOD1-3* family and the *NOX1-5* family from human immortalized Nthy thyroid cells modeling a normal thyroid and from papillary thyroid cancer patient-derived TPC1 cells. Comparable to our previous data^26^, we observed increased *SOD2, NOX1, NOX2*, and *NOX5* mRNA expression in papillary thyroid cancer cells compared to Nthy cell line. Importantly, we found decreased expression of *SOD3* recurrently with recent works^23^,^26^ and an absence of *NOX4* expression (Fig. 4A).

The redox gene expression patterns of Thyroid MSCs compared to PTC MSCs were similar to the expressions observed in thyroid epithelial Nthy and TPC1 cells, with the exceptions of *SOD3* and *NOX4*. The *NOX4* gene was undetectable in the epithelial Nthy and TPC1 cells, whereas Thyroid MSCs and PTC MSCs both had high *NOX4* expression levels. Analysis of *SOD3* mRNA demonstrated increased expression in PTC MSCs compared to in Thyroid MSCs, thus suggesting autocrine-paracrine conversion of *SOD3* mRNA synthesis (Fig. 4B). To confirm the increased *SOD3* levels in the PTC MSCs, we analyzed the mRNA expression of the enzyme from each patient and determined the stimulatory effect of TPC1 cancer cell condensed medium on *SOD3* mRNA levels. Interestingly, the mRNA production was significantly increased in all five samples of patient-derived PTC MSCs compared to their Thyroid MSC counterparts (Fig. 4C–G), which was shown to be enhanced by TPC1 paracrine action (Fig. 4F,G).

### Paracrine MSC secreted *SOD3* increases epithelial cancer cell growth

**Figure 5:**
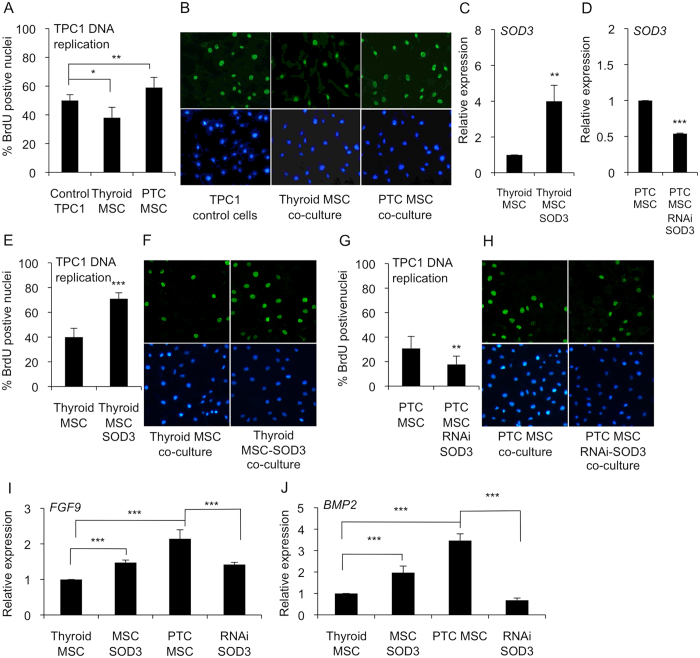
Paracrine effect of SOD3 on TPC1 cancer cell growth. (**A**,**B**) TPC1 cell growth in Thyroid MSC and PTC MSC co-cultures suggested significantly decreased TPC1 DNA replication in the Thyroid MSC culture and significantly increased DNA replication in the PTC MSC co-culture. (**C**) *SOD3* expression in Thyroid MSCs and Thyroid MSCs transduced with *SOD3* lentivirus. (**D**) *SOD3* expression in PTC MSCs and PTC MSCs transduced with RNAi *SOD3* lentivirus. (**E**,**F**) TPC1 DNA replication in Thyroid MSCs and Thyroid MSCs transduced with *SOD3* lentivirus suggested increased SOD3-driven TPC1 growth. (**G**,**H**) RNA interference of *SOD3* in PTC MSCs suggested decreased TPC1 DNA replication correlated with decreased *SOD3* expression. (**I,J**) *FGF9* and *BMP2* gene expression analysis suggested increased SOD3-driven expression in Thyroid MSCs transduced with *SOD3* lentivirus and decreased growth factor expression in PTC MSCs transduced with RNAi *SOD3* lentivirus compared to controls. The p-values (*p < 0.05, **p < 0.01, ***p < 0.001) were determined by two-tail independent samples t-tests.

According to previous data, BM MSCs could reduce cancer cell proliferation via the paracrine system, thereby inhibiting tumorigenesis^32^. On the contrary, tumor-associated stromal cells, such as tumor-associated fibroblasts and mesenchymal stem cells, have been shown to promote cancer cell proliferation and tumor growth^33^,^34^. Therefore, we tested the growth stimulating properties of Thyroid MSCs and PTC MSCs on the papillary thyroid cancer TPC1 cell line. In line with previous reports, a BrdU DNA incorporation assay for TPC1 papillary thyroid cancer cells cultured together with Thyroid MSCs suggested decreased DNA replication, whereas cancer cells grown in the presence of PTC MSCs showed significantly increased BrdU incorporation (Fig. 5A,B).

To probe the paracrine effect of *SOD3* expression on cancer cell growth, we over-expressed *SOD3* in Thyroid MSCs (Thyroid MSC SOD3) and silenced the gene expression by *SOD3 RNAi* in PTC MSCs (PTC MSC SOD3 RNAi) (Fig. 5C,D). Co-culture of TPC1 cells with Thyroid MSC SOD3 suggested significantly (p < 0.01) increased DNA replication of TPC1 cells compared to the parental Thyroid MSCs (Fig. 5E,F). Correspondingly, RNAi *SOD3* treatment of PTC MSCs decreased (p < 0.001) TPC1 DNA replication compared to parental PTC MSCs (Fig. 5G,H), thus demonstrating that PTC MSCs may support cancer cell growth by secreting SOD3.

SOD3 has been demonstrated to regulate the activation of a large number of growth-related signaling molecules^14^,^27^. We then studied the growth factor gene expression in Thyroid MSC SOD3 and PTC MSC SOD3 RNAi cells. The analysis revealed correlations among *SOD3, FIBROBLAST GROWTH FACTOR 9 (FGF9*), and *BONE MORPHOGENIC PROTEIN 2 (BMP2*) expression (Fig. 5I,J).

### Paracrine effect of *SOD3* on epithelial cancer cell migration

**Figure 6:**
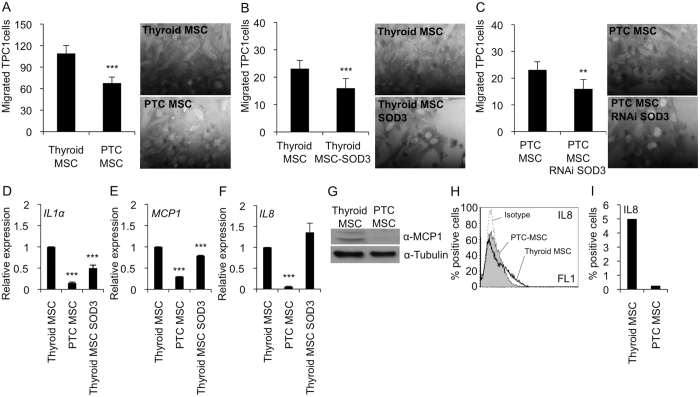
Effect of SOD3 on TPC1 cancer cell migration. (**A**) TPC1 cancer cells migrated significantly faster towards Thyroid MSCs than PTC MSCs. Representative high-power fields from Matrigel shown. (**B**) Increased *SOD3* expression in Thyroid MSCs significantly decreased TPC1 cell migration. Representative high-power fields from Matrigel are shown. (**C**) Reduced *SOD3* expression reduced TPC1 cell migration toward PTC MSCs. Representative high-power fields from Matrigel are shown. (**D**) *IL1α* expression was significantly decreased in the PTC MSCs compared to the Thyroid MSCs. SOD3 over-expression in Thyroid MSCs significantly reduced *IL1α* mRNA synthesis compared to the Thyroid MSC control. (**E**) *MCP1* mRNA expression was significantly decreased in PTC MSCs compared to Thyroid MSCs. SOD3 over-expression in the Thyroid MSCs significantly reduced *MCP1* mRNA synthesis compared to the Thyroid MSC control. (**F**) *IL8* mRNA expression was significantly decreased in the PTC MSCs compared to the Thyroid MSCs. SOD3 over-expression in the Thyroid MSCs did not affect *IL8* expression. (**G**) MCP1 Western blot confirmed decreased *MCP1* mRNA expression in the PTC MSCs compared to the Thyroid MSCs. (**H**) Flow cytometer analysis of IL8 expression confirmed decreased *IL8* mRNA expression in the PTC MSCs compared to Thyroid MSCs. (**I**) Percentage of positive cells in the IL8 flow cytometer analysis. The p-values (*p < 0.05, **p < 0.01, ***p < 0.001) were determined by two-tail independent samples t-tests.

Desmoplastic thyroid tumors frequently metastasize to the lymph nodes^29^, suggesting increased intratumoral cancer cell migration toward the lymphatic vasculature. In the current work, the Matrigel migration analysis demonstrated significantly (p < 0.001) increased TPC1 migration levels towards Thyroid MSCs compared to PTC MSCs (Fig. 6A), which may indicate a lower affinity of TPC1 cells to tumor stroma. The analysis of the effect of SOD3 on TPC1 cell migration showing significantly (p < 0.01) reduced migration toward *SOD3* over-expressing Thyroid MSCs (Thyroid MSC SOD3) (Fig. 6B) could suggest regulation of local cancer cell migration by paracrine secretion of the enzyme. However, silencing of *SOD3* in PTC MSCs (PTC MSC SOD3 RNAi) failed to show increased cancer cell migration (Fig. 6C), indicating that SOD3 might have a different function in fibrotic MSCs or that the activation process of PTC MSCs modifies the ROS balance, hence altering the effect of single redox genes.

Previous publications have proposed that inflammatory and cancer cell migration are regulated by the same chemoattractants^35^,^36^. We recently demonstrated SOD3-derived decreased expression of *TNFα, IL1α, IL6, MIP2*, and *MCP-1* inflammatory cytokines in cardiovascular models with consequent inhibition of monocyte/macrophage migration^17^,^20^,^37^. The expression analysis of *IL1α, MCP-1*, and *IL8* showed reduced cytokine expression in PTC MSCs compared to Thyroid MSCs (Fig. 6D–J). The data, however, suggest that only *IL1α* and *MCP-1* are downregulated by SOD3 in stromal cells (Fig. 6D,E), whereas *IL8* expression is independent of the enzyme (Fig. 6F). Western blot analysis for MCP-1 (Fig. 6G) and flow cytometer analysis for IL8 (Fig. 6H,I) supported the mRNA expression data showing decreased expression in PTC MSCs compared to Thyroid MSCs. Our current data thus support our previous observations demonstrating that SOD3 stimulates cell proliferation and inhibits cell migration^17^,^18^,^20^,^23^,^24^,^25^,^26^,^27^,^28^,^37^,^38^.

## Discussion

Local vascular permeability with consequent extravasation of fibrinogen and plasminogen initiate the formation of fibrin gel deposits, the early form of tumor stroma, which attracts migrating fibroblasts, epithelial cancer cells and inflammatory cells. Importantly, continuous tumor growth creates a demand for continuous stroma expansion. Thus, tumor stroma has different developmental stages that affect cancer cells, regulating their proliferation and local migration. According to recent data, the development of stroma is connected to the progression of carcinogenesis, such as lymph node metastasis^39^, indicating that stroma responds to the microenvironmental needs of epithelial cancer cells through paracrine growth factor secretion and direct cell-to-cell contact^40^,^41^,^42^. Additionally, activated desmoplastic stroma, which is frequently observed in medullary thyroid cancers, is used as a clinical intraoperative diagnostic marker to characterize tumor progression and to predict lymph node metastasis^29^,^39^,^43^,^44^. In the present work, we observed a marked inverse correlation between desmoplasia and inflammatory cell infiltration in PTC and FTC: 12 out of 20 patients had increased desmoplasia or increased mononuclear cell infiltration, whereas only two out of 20 patients had simultaneous desmoplasia and inflammation (Fig. 1). Previous articles have suggested that aggressive cancers, such as pancreatic ductal adenocarcinoma characterized by an excessive stromal reaction, may have reduced intratumoral vascularization^45^ that could cause reduced mononuclear cell migration into desmoplastic tumors. However, in a recent work the immunohistochemical analysis of fibroblast activating protein α-positive medullary thyroid carcinomas showed no correlation between new vessel formation and stromal fibrosis^29^, thus indicating other mechanisms than the physiological fibrotic barrier in the inhibition of cell migration. Inflammation has also been suggested to precede fibrosis development: infiltrated inflammatory cells secrete profibrogenic cytokines that affect fibroblast activation, perivascular fibrosis development, and cellular migration^46^,^47^.

An interesting, although rare, characteristic of thyroid tumors is intratumoral heterotopic ossification^48^,^49^,^50^,^51^,^52^,^53^,^54^, which may suggest the presence of multipotent mesenchymal stem/stromal cells in thyroid tissue. Indeed, tumor-associated MSCs recently characterized in ovarian carcinoma and prostate cancer have similar phenotype and functionality as BM MSC but altered growth factor expression profile^55^,^56^. The current data, which demonstrate the presence of MSCs in non-carcinogenic thyroid and papillary thyroid cancer with a similar differentiation capacity and CD marker expression as BM MSCs (Fig. 2) strengthening the previous observations, could further indicate that MSCs maintain their stemness even in conditions where they are under constant external carcinogenic stimuli. However, a more accurate gene expression analysis of fibrotic markers in Thyroid MSCs and PTC MSCs (Fig. 3) showed differentiation stage-related differences that affected functional properties, such as proliferation (Fig. 5A) and migration (Fig. 6A), in neighboring cells. We demonstrated that PTC MSCs had increased expression of fibrotic markers (Fig. 3). Although MSC cultures in general are heterogeneous containing cells with various degrees of differentiation, the current data may suggest that tumor stroma MSCs exposed to cancer cell secretion could obtain similar phenotypic characteristics as cancer associated fibroblasts and therefore serve as an origin of cells for activated fibroblasts.

ROS have been demonstrated to contribute to carcinogenesis and myofibroblast differentiation. Interestingly, *NOX4* expression, which is absent in thyroid cancer cells^26^ (Fig. 4), was observed in MSCs, which may suggest a paracrine role for the protein in the thyroid. Furthermore, *SOD3* expression suggested autocrine-paracrine conversion from epithelial cancer cells to mesenchymal stromal cells (Fig. 4), which could shed light on the function of SOD3 in tumorigenesis. Previous data have shown increased *SOD3* expression in an experimental rat thyroid goiter model^23^ with corresponding growth-supporting function at physiological expression levels^14^,^18^,^25^,^26^,^28^,^57^. A strong connection of SOD3 to growth regulation is caused by positive a feedback loop between the enzyme, small GTPase activation, and RAS-ERK1/2 signaling^24^,^25^,^27^,^28^. Moderately increased small GTPase RAS activation stimulates *SOD3* mRNA synthesis, but at aberrant levels of RAS activity (>10-fold RAS activation level), there is a sudden decrease in *SOD3* expression^24^. Therefore, we have hypothesized that SOD3 benefits the initial phase of tumorigenesis at low (<10-fold) RAS activation levels. Once SOD3 expression reaches non-physiological toxic levels, cancer cells are programmed to downregulate autocrine SOD3 production and instead stimulate stromal paracrine SOD3 secretion simultaneously with activation of stroma, as shown in Figs 3 and 4. Paracrine secretion of SOD3 then continues to support cancer cell growth (Fig. 5) and, correspondingly, decreases the affinity of cancer cells to tumor stroma by decreasing chemotactic cytokine expression (Fig. 6), allowing local intratumoral cancer cell migration.

In conclusion, based on the current data, PTC MSCs with a fibrotic fingerprint might function as a source of myofibroblasts. Importantly, clarification of the mechanisms of fibroblast activation could result in the discovery of novel drug targets that are specific to thyroid cancer^58^ or cancer stroma. ROS, although forming a challenging molecular ensemble, control growth- and migration-related signaling routes that potentially contain drug targets. The current results suggest the autocrine-paracrine conversion of *SOD3* expression, corroborating the growth supportive nature of the enzyme throughout the carcinogenic process. Thus, the identification of SOD3-coordinated signal transduction in tumor stroma and epithelial cancer cells may reveal small drug target molecules that could be used in combination therapy for the treatment of thyroid cancer.

## Methods

### Histological analysis

For histological analyses, papillary and follicular thyroid carcinoma samples were fixed in formalin, embedded in paraffin, and cut into 4–5 μm sections with a microtome. Sections were de-waxed in xylene for 10 minutes, rehydrated by sequential immersion in decreasing ethanol concentrations. The sections were then stained with hematoxylin-eosin and analyzed using an optical microscope (Nikon ECLIPSE Ni, Nikon Instrument Europe B.V. Amsterdam, Netherlands). Two independent observers (F.C. and F.R.) examined the specimens.

### Isolation and culture of stromal cells

Thyroid mesenchymal stem/stromal cells (MSCs) were isolated from non-malignant sites (Thyroid MSCs) and papillary thyroid cancer sites (PTC MSCs) of patients. De-identified thyroid tissue samples were obtained from patients who underwent thyroid surgery at the University of Wisconsin-Madison. The protocol was approved by the Health Sciences Institutional Review board (IRB) of the University and performed in accordance with the relevant guidelines and regulations^59^. Informed consent was obtained from all subjects according the requirements of University of Wisconsin-Madison protocol permission. Due to differences between individuals all analysis of the study were done comparing thyroid MSCs and PTC-MSCs of the same patient. Tumor tissue was washed several times with PBS, cut into small pieces, and then was plated in alpha MEM (Mediatech, Manassas, VA, USA) supplemented with 10% FBS (Hyclone, Logan, UT), non-essential amino acids (Mediatech) and L-alanine-l-glutamine (Life Technologies, Grand Island, NY, USA). After reaching 70–90% confluency, cells were harvested using TrypLE (Life Technologies) and passaged until reaching passage 4 before characterizing and using them in the study^60^. The MSCs were transduced with SOD3 and RNAi SOD3 lentivirus (MOI 10) (Dharmacon, Lafayette, Colorado, United States).

### Differentiation assay

Cells seeded in dishes were stimulated with 10^−6^ M dexamethasone, 100 *μ*g/ml 3-isobutyl-1-methylxanthine, 50 *μ*M indomethacin, and 10 *μ*g/ml insulin (Sigma) for adipogenic differentiation, with 1 ng/ml recombinant human transforming growth factor-*β*1 (TGF-*β*1) (Sigma) for chondrogenic differentiation and with 10^−7^ M dexamethasone, 10 mM *β*-glycerophosphate disodium, and 50 *μ*g/ml ascorbic acid (Sigma) for osteogenic differentiation. Adipogenic differentiation was analyzed after 0.3% oil red O (Sigma) treatment, chondrogenic differentiation was analyzed after anti-human type II collagen MAb treatment, and osteogenic differentiation was analyzed after alizarin red S (Sigma) treatment^60^,^61^.

### Flow cytometer assay

For the flow cytometry analysis, 100 000 cells were incubated in a 100 μl volume with antibodies on ice. The cells were washed twice with PBS, resuspended in 500 μl, filtrated, and analyzed.

### Gene expression analysis

RNA was isolated using an RNeasy minikit (Qiagen, Hilden, Germany). The cDNA synthesis was performed by QuantiTect Reverse Transcription (Qiagen). SYBR Green PCR master mix (Applied Biosystems, Foster City, CA, USA) was used for qPCR. The primers are listed in the Supplementary Table T1.

### Western blot analysis

The cells were homogenized in lysis buffer (50 mmol/L HEPES pH 7.5, 150 mmol/L NaCl, 10% glycerol, 1% Triton X-100, 1 mmol/L EGTA, 1.5 mmol/L MgCl_2_, 10 mmol/L NaF, 10 mmol/L sodium pyrophosphate, 1 mmol/L Na_3_VO_4_, 10 μg approtinin/ml, and 10 μg leupeptin/ml) (Sigma). The antibodies used were αMCP1 (Abcam, Cambridge, UK) and α-tubulin (Cell Signaling, Danvers, MA, USA).

### Growth analysis

To characterize DNA replication, 10 mM bromodeoxyuridine (BrdU) (Roche, Basel, Switzerland) was added to the growth medium for 15 min. Subsequently, the cells were washed three times with PBS, fixed in 3% paraformaldehyde (Sigma) for 20 minutes at −20 °C, washed three times with PBS, and stained using anti-BrdU antibody (Roche) primary antibody, FITC–conjugated secondary antibody (Jackson ImmunoResearch Laboratories Inc., West Grove, PA, USA), and Hoechst nuclear stain (Sigma).

### Migration analysis

For the migration assay, 100 μl of Matrigel (Corning, Corning, NY, USA) at 1 mg/ml was added to the migration chamber (8 microns, BD, San Jose, CA, USA) and allowed to stabilize at room temperature for 30 minutes. The chambers were moved to MSC cultures into 12-well plates, 50 000 TPC1 cells were added to the Matrigel and allowed to migrate for 24 hours at 37 °C. After incubation, the Matrigel was removed from the chamber, and the migrated cells were fixed with 7% paraformaldehyde (Sigma), washed with PBS, and stained with Cristal violet (Sigma). The migrated cells were counted from the high-power microscope fields.

### Statistical analyses

The experiments were repeated at least three times. All results are expressed as the mean ± SD. The p-values (*p < 0.05, **p < 0.01, ***p < 0.001) were determined by two-tail independent samples t-tests.

## Additional Information

**How to cite this article:** Parascandolo, A. *et al*. Extracellular Superoxide Dismutase Expression in Papillary Thyroid Cancer Mesenchymal Stem/Stromal Cells Modulates Cancer Cell Growth and Migration. *Sci. Rep.* **7**, 41416; doi: 10.1038/srep41416 (2017).

**Publisher's note:** Springer Nature remains neutral with regard to jurisdictional claims in published maps and institutional affiliations.

## Supplementary Material

Supplementary Information
