# Supplementary material for: Extracellular Superoxide Dismutase Expression in Papillary Thyroid Cancer Mesenchymal Stem/Stromal Cells Modulates Cancer Cell Growth and Migration
Source: Sci Rep. 2017 Feb 20;7:41416. doi: 10.1038/srep41416 (PMC5316948; doi:10.1038/srep41416)
Supplement: Supplementary Information [file srep41416-s1.pdf]

Extracellular Superoxide Dismutase Expression in Papillary Thyroid Cancer Mesenchymal Stem/Stromal Cells Modulates Cancer Cell Growth and Migration

Alessia Parascandolo[1], Francesca Rappa[2,3], FrancescoCappello[2,3], Jaehyup Kim[4], David A. Cantu[4], HerbertChen[5], Gianluigi Mazzoccoli[6], Peiman Hematti[4,7], Maria DomenicaCastellone[8,9], Marco Salvatore[1] & Mikko O. Laukkanen[1]

Table T1

	Forward primer	Reverse primer
FAP	tacgtttcatcactggccct	catctgctgttccgtggatg
Col1A1	gctactaccgggctgatgat	accagtctccatgttgcaga
Tenascin	acaacatcaagctgccagtg	ggggatgttgatgcgatgtg
SOD1	agggcatcatcaatttcgag	acattgcccaagtctccaac
SOD2	tgtaccggttccgagttttc	ttcaggccctacaattcacc
SOD3	cttcgcctctgctgaagtct	gggtgtttcggtacaaatgg
NOX1	ttaacagcacgctgatcctg	ctggagagaatggaggcaag
NOX2	tcacttcctccaccaaaacc	gggattgggcattcctttat
NOX3	gcccaactggaacaatgagt	ttcctggtggagttctttgg
NOX4	cttccgttggtttgcagatt	tgggtccacaacagaaaaca
NOX5	ctacgtggtagtggggctgt	atgcaggaactggagcagat
FGF9	gtggactctacctcgggatg	gtgtgaatttctggtgccgt
BMP2	aatgcaagcaggtgggaaag	gctgtgttcatcttggtgca
IL1α	tgatcagtacctcacggctg	tggtcttcatcttgggcagt
MCP1	caatcaatgccccagtcacc	ggtggtccatggaatcctga
IL8	cagttttgccaaggagtgct	acttctccacaaccctctgc
β-actin	gacattaaggagaag	gctcgtagctcttctcca
